# Analysis of the Origin and Evolutionary History of HIV-1 CRF28_BF and CRF29_BF Reveals a Decreasing Prevalence in the AIDS Epidemic of Brazil

**DOI:** 10.1371/journal.pone.0017485

**Published:** 2011-03-01

**Authors:** Natalia Ristic, Jean Zukurov, Wagner Alkmim, Ricardo Sobhie Diaz, Luiz Mario Janini, Mario P. S. Chin

**Affiliations:** 1 Aaron Diamond AIDS Research Center, The Rockefeller University, New York, New York, United States of America; 2 Department of Medicine, Federal University of Sao Paulo, Sao Paulo, Brazil; 3 Department of Microbiology, Immunology, and Parasitology, Federal University of Sao Paulo, Sao Paulo, Brazil; Institut Pasteur, France

## Abstract

**Background:**

HIV-1 subtype B and subtype F are prevalent in the AIDS epidemic of Brazil. Recombinations between these subtypes have generated at least four BF circulating recombinant forms (CRFs). CRF28_BF and CRF29_BF are among the first two BF recombinants being identified in Brazil and they contributed significantly to the epidemic. However, the evolution and demographic histories of the CRFs are unclear.

**Methodology/Principal Findings:**

A collection of *gag* and *pol* sequences sampled within Brazil was screened for CRF28_BF-like and CRF29_BF-like recombination patterns. A Bayesian coalescent framework was employed to delineate the phylogenetic, divergence time and population dynamics of the virus having CRF28_BF-like and CRF29_BF-like genotype. These recombinants were phylogenetically related to each other and formed a well-supported monophyletic clade dated to 1988–1989. The effective number of infections by these recombinants grew exponentially over a five-year period after their emergence, but then decreased toward the present following a logistic model of population growth. The demographic pattern of both recombinants closely resembles those previously reported for CRF31_BC.

**Conclusions:**

We revealed that HIV-1 recombinants of the CRF28_BF/CRF29_BF clade are still circulating in the Brazilian population. These recombinants did not exhibit a strong founder effect and showed a decreasing prevalence in the AIDS epidemic of Brazil. Our data suggested that multiple URFs may also play a role in shaping the epidemic of recombinant BF HIV-1 in the region.

## Introduction

HIV-1 is classified into Group M, O, N and P. Group M HIV-1 dominates the AIDS pandemic with at least nine subtypes and multiple intersubtype recombinants currently identified [1]. The intersubtype recombinants are the result of recombination among the subtypes of HIV-1. Furthermore, these intersubtype recombinants can also recombine with HIV-1 of the same or different subtypes or with other recombinants to generate more complex recombinants. They emerge in almost every region of the world where more than one HIV-1 subtype is present. Currently, 48 circulating recombinant forms (CRFs) and a large number of unique recombinant forms (URFs) have been identified. These CRFs and URFs accounted for almost 18% of new infections in 2004 [2] and they continue to play an increasingly important role in shaping the AIDS pandemic.

It is reported that there are more than 545,000 AIDS cases in Brazil [3]. The epidemic is particularly severe in the southeastern part of the country, where large urban centers such as Rio de Janeiro and Sao Paulo are situated. At the end of 2008, the southeastern states (Espirito Santo, Minas Gerais, Rio de Janeiro and Sao Paulo) reported at least 323,000 diagnosed AIDS cases (59.3% of all cases in the country) with approximately 19.2 AIDS cases per 100,000 people [3]. The epidemic in the southeastern region of the country is complicated by multiple subtypes and recombinants of HIV-1 circulating in the population. For instance in the city of Sao Paulo, the major subtypes are subtype B (79%–88%) and subtype F (4%–11%) [4], [5]. Such a dense transmission network, with a high HIV-1 incidence rate and multiple subtypes present in the region, provides a perfect breeding ground for new HIV-1 recombinants. Indeed, HIV-1 recombinants between subtype B and subtype F have been frequently identified since the introduction of subtype F in Brazil [6], [7], [8], [9], [10], [11], [12]. Studies have shown that up to 9% of the HIV-1 isolated in Sao Paulo have a mosaic B/F *pol* genome [4], [5]; in particular, the CRF28_BF and CRF29_BF recombinants were identified in the region in 1999 [13], [14].

**Figure 1:**
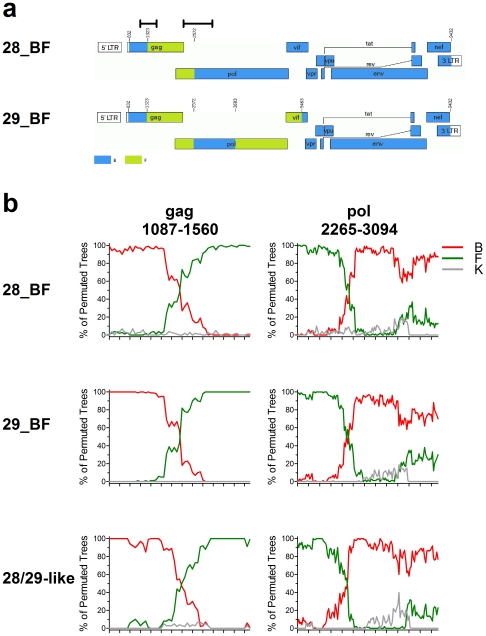
Mapping the recombination breakpoints of CRF. (A) Recombination patterns of the CRF28_BF and CRF29_BF genomes. The number at each recombination breakpoint indicates the nucleotide position according to HXB2 numbering. Blue, subtype B; Green, subtype F1. Black bars above the HIV-1 genome indicate the *gag* (1,087–1,560) and *pol* (2,265–3,904) regions analyzed in this study and in the subsequent bootscanning analyses. (B) The bootscanning of CRF28_BF, CRF29_BF and CRF28/29_BF-like recombinant genomes revealed a similar recombination breakpoint. The recombinant sequences identified in RIP were subjected to bootscanning, and representative results are shown. The query sequence was compared with subtype B, F1 and K consensuses obtained from RIP, and plots of percentages of permuted trees against nucleotide positions are shown. Red, subtype B; green, subtype F1, grey, subtype K. A window/step size of 200/10 was used to analyze the sequences.

CRF28_BF and CRF29_BF were identified in patients at the Counseling and Testing Centers in Santos, which is a port city located 80 km from the city of Sao Paulo [14]. CRF29_BF were also identified in patients from Sao Paulo and Rio de Janeiro [6], [13]. These CRFs are genetically distinct from CRF12_BF, which was first detected and subsequently found to be widely circulating in Argentina [15], [16], [17]. The majority of the CRF28_BF and CRF29_BF genome belongs to subtype B and is closely related to the subtype B HIV-1 circulating in Brazil (Figure 1A) [14]. The subtype F regions are phylogenetically linked to the Brazilian subtype F1. CRF29_BF and CRF28_BF share similar recombination breakpoints in the *gag* and *pol* genes at nucleotide (nt) positions 1,322 and 2,571 (HXB2 numbering). CRF29_BF has an additional subtype F region spanning from nt 3,682 to 5,462, creating additional breakpoints in the *pol* and *vif* genes. Owing to the common recombination breakpoints that the recombinants share, it is possible that a common ancestor of the BF recombinants emerged early in the epidemic and has evolved through subsequent recombination events.

Although several studies have been carried out to characterize the epidemiology and phylogenetics of subtype B and subtype F HIV-1, and BF URFs in Brazil [4], [5], [6], [7], [8], [9], [10], [11], [13], [18], little is known about the evolutionary and demographic histories, and the epidemic potential of the CRF28_BF and CRF29_BF. Here, we reconstructed the evolutionary history of the CRF28_BF and CRF29_BF by coalescent inference to delineate their origins and population dynamics. Our data showed that since the emergence of the CRF28_BF and CRF29_BF in the late 1980s, both CRFs underwent rapid population growth; however, the growth rate has slowed in recent years. Both CRFs did not exhibit a strong founder effect, which suggested that multiple URFs may also play a role in shaping the recombinant BF HIV-1 epidemic in the region. Our study provides important insights into the contributions of HIV-1 recombinants to the AIDS epidemic in South America.

## Methods

### Study population and data preparation

Two regions of the HIV-1 genome encompassing the recombination breakpoints in *pol* (nt position 2,572) and *gag* (nt position 1,323) of CRF28_BF and CRF29_BF were analyzed (Figure 1A) [13], [14]. For the *pol* region, we studied a set of 111 HIV-1 sequences obtained from patients attending HIV/AIDS treatment centers in Sao Paulo [13]. An 830-bp-long *pol* region covering part of the protease and reverse transcriptase genes (nt positions 2,265–3,094) was sequenced as previously described [13]. Sequences were analyzed together with 433 subtype B, 61 recombinant BF, three CRF28_BF and four CRF29_BF sequences isolated in Brazil with known sampling dates retrieved from the Los Alamos HIV Sequence Database. This resulted in a dataset consisting of 612 *pol* sequences collected between 1989 and 2007. Similarly, we obtained 184 HIV-1 *gag* sequences isolated in Brazil with known sampling dates from the Los Alamos HIV Sequence Database, covering parts of the matrix and capsid genes (nt positions 1,087–1,560). This dataset consisted of 127 subtype B, 50 recombinant BF, three CRF28_BF and four CRF29_BF sequences collected between 1989 and 2006. The nucleotide sequences were aligned using Clustal X [19], and alignment gaps were excluded from the analyses.

### Identification of the CRF28/29_BF-like HIV-1

The workflow for identifying recombination in HIV-1 was described previously [20]. The sequences in the *pol* and *gag* datasets were first analyzed using the Recombination Identification Program (RIP) 3.0 of the Los Alamos HIV Sequence Database to identify subtype B and CRF28/29_BF-like sequences. In the analysis, each individual query sequence was compared with subtype B and subtype F1 consensuses using a window size of 200 bp. Any query sequence that had a value of 0.9 or higher in the s-distance with the subtype B consensus, free of recombination breakpoints, was classified as subtype B. The sequences having a recombination pattern identical to those of CRF28_BF and CRF29_BF were regarded as CRF28/29_BF-like. The recombinant sequences were compared with the subtype B, subtype F1 and subtype K consensus sequences obtained from RIP in a bootscanning analysis using SimPlot to confirm their genetic identity [21]. Based on the analyses, we then compiled a refined dataset consisting of *pol* sequences of a selection of subtype B and all of the identified CRF28/29_BF-like, three CRF28_BF and four CRF29_BF sequences with a compromise between wide sampling date and a realistic size of the dataset. A refined dataset with the *gag* sequence was also constructed using the same approach.

### Bayesian evolutionary analysis by sampling phylogenetic trees of subtype B and CRF28/29_BF-like HIV-1

We performed phylogenetic analysis on the two refined datasets of *pol* and *gag* sequences using a Bayesian approach with the SRD06 nucleotide substitution model [22]. The purpose of this phylogenetic analysis was to identify the overall topology and confirm the relationship between the subtype B and the CRF28/29_BF-like HIV-1. A Bayesian Markov Chain Monte Carlo (MCMC) analysis, as implemented in the program BEAST v1.5.4 [23], was carried out for 10 million generations to produce 10,000 trees for each dataset. The maximum clade credibility phylogenetic tree was determined in TreeAnnotator v1.5.4 after excluding an initial 10% of the sample.

### Estimation of nucleotide substitution rate, demographic history and time of divergence

We used an MCMC method, as implemented in the program BEAST v1.5.4 [23], to estimate the rate of nucleotide substitution. The refined *pol* and *gag* datasets were analyzed with the codon-based SRD06 nucleotide substitution model [22]. Three clock models were used in the analyses: the strict clock model assumes a single evolutionary rate for all the lineages; and the uncorrelated exponential relaxed clock and the uncorrelated lognormal relaxed clock allow evolutionary rates to vary among lineages within exponential and lognormal distributions, respectively [24]. These three clock models were statistically compared for each dataset using a Bayes Factor test to find the best fit [25]. We used the best fit clock model to estimate a posterior distribution for the rate of nucleotide substitution and then used as an empirical prior distribution in the coalescent analyses that follow.

The CRF28/29_BF-like sequences were analyzed using a nonparametric model (Bayesian skyline plot) to estimate the change in the effective population size through time and to infer demographic information within the lineage [26]. Four demographic models were compared to select the model that best described the epidemiological history of the CRF28/29_BF-like HIV-1. The models tested in this study were as follows: constant population size, exponential growth, logistic growth and expansion growth [27]. The Bayes Factor test was used to evaluate the demographic models for each dataset [25]. We performed three independent Bayesian MCMC runs for 10 million generations to produce 10,000 trees for each analysis. The convergence of parameters was assessed through the effective sampling size, with all parameters for each run having values >100 indicating a sufficient level of sampling. The mean time to the most recent common ancestor (TMRCA) and the maximum clade credibility of the phylogenetic trees were then calculated after the removal of 10% of the samples following visual inspection in the programs Tracer v1.5 and FigTree v1.3.1.

Recombination between CRF28/29_BF-like recombinants and the recombination breakpoints in the *gag* and *pol* sequences may cause biases in the estimation of nucleotide substitution rate and TMRCA [28], [29]. To test the possible effects of recombination on the current analysis, we employed an unlinked multilocus model in BEAST, which allows independent coalescent pathways for different gene loci [30]. In this model, the linked recombinant *pol* gene was divided into two unlinked loci of subtype F (nt positions 2,265–2,572) and subtype B (nt positions 2,573–3,094) sequences. The nucleotide substitution rates, demographic histories and times of divergence of the two unlinked loci were estimated in the Bayesian coalescent framework. Similar approach was applied to the subtype B loci (nt positions 1,087–1,323) and subtype F loci (nt positions 1,324–1,560) of the recombinant *gag* gene.

## Results

### Identification of CRF28/29_BF-like recombination breakpoints in HIV-1

We analyzed the 612 sequences of the *pol* dataset using RIP to identify recombination breakpoints similar to those of CRF28_BF and CRF29_BF. Of the 433 subtype B *pol* sequences in the dataset, we found four sequences retrieved from the HIV Sequence Database that had recombination breakpoints similar to those of CRF28/29_BF (Figure 1B). The accession numbers of these four sequences are AF112891, AF112898, AF112953 and AY213549. In addition, we identified CRF28/29_BF-like recombination breakpoints in the *pol* gene of two sequences from the 111 patient samples sequenced in this study. Among the 61 recombinant BF sequences from the HIV Sequence Database, 21 of them possessed CRF28/29_BF-like recombination breakpoints. Based on the recombination breakpoint analysis, we compiled a refined *pol* dataset with 102 sequences consisting of 66 Brazilian subtype B, 27 CRF28/29_BF-like, 3 CRF28_BF and 4 CRF29_BF sequences sampled between 1989 and 2007.

The 184 sequences of the *gag* dataset were analyzed using a similar approach. We found that there were three sequences (U86560, AY071973 and AY071985) in the 127 subtype B sequences that had CRF28/29_BF-like recombination breakpoints (Figure 1B). We also identified 32 CRF28/29_BF-like recombinant sequences from the 50 BF recombinant sequences. We then compiled a refined dataset of 90 *gag* sequences containing 48 Brazilian subtype B, 35 CRF28/29_BF-like, three CRF28_BF and four CRF29_BF sequences sampled between 1989 and 2006.

### Bayesian phylogeny of Brazilian subtype B and CRF28/29_BF-like HIV-1

**Figure 2:**
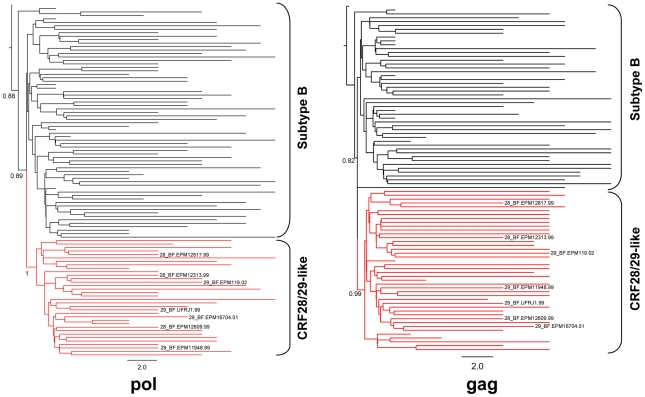
Bayesian evolutionary analysis by sampling phylogenetic trees of Brazilian subtype B and CRF28/29_BF-like HIV-1. The maximum clade credibility phylogenetic trees of the *pol* and *gag* genes are shown. The brackets indicate the monophyletic group formed by each lineage. The subtype B lineage is in black, and the CRF28/29_BF-like lineage is in red. Positions of the CRF28_BF and CRF29_BF references are indicated. Posterior probability values greater than 0.8 are shown at nodes.

A total of 102 *pol* sequences, consisting of 66 subtype B and 27 CRF28/29_BF-like HIV-1, were aligned with the reference sequences of CRF28_BF and CRF29_BF. A Bayesian analysis was used to infer the phylogenetic relationships of the isolates. The resulting tree showed that the CRF28/29_BF-like sequences form a well-supported monophyletic group (posterior probability = 1) distinct from the subtype B sequences (Figure 2). All of the sequences in the CRF28/29_BF-like lineage have recombination breakpoints similar to those of CRF28_BF and CRF29_BF (Figure 1B). Remarkably, the CRF28_BF and the CRF29_BF references did not form a distinct group, and both references were scattered in different branches of the CRF28/29_BF-like lineage, suggesting that the two strains share a common ancestry.

The Bayesian analysis of the refined dataset of *gag* sequences, which consisted of 48 subtype B, 35 CRF28/29_BF-like, three CRF28_BF and four CRF29_BF sequences, revealed a similar topology to the *pol* phylogenetic tree (Figure 2). The CRF28/29_BF-like sequences formed a distinct lineage with high support, and all of the sequences within had a similar recombination profile as the reference CRF28_BF and CRF29_BF (Figure 1B). The phylogenies inferred from the *gag* and *pol* datasets indicated that CRF28_BF and CRF29_BF are closely related and have co-evolved since their emergence.

### Estimation of the evolution rate of the Brazilian CRF28/29_BF-like HIV-1

Bayesian MCMC analyses under a skyline tree prior were used to estimate the rate of evolution of the Brazilian CRF28/29_BF-like HIV-1. We first evaluated the marginal likelihood of the three clock models employed in this study to determine the best clock model for estimating the evolution rate of the *pol* gene. Analysis of the Bayes Factor showed that the model using a lognormal relaxed clock fit the dataset better than the models assuming an exponential relaxed clock or a strict clock (Table S1). Under the lognormal relaxed clock model, the estimated mean rate of evolution of the *pol* gene was 2.60×10^−3^ substitutions per nucleotide site per year (Table 1).

**Table 1:** Population dynamics estimates for the CRF28/29_BF epidemic.^a^

Gene	μ, site^−1^ year^−1^	Origin of the lineage	Number of effective infections	*r*, year^−1^	λ, month
*pol*					
Linked locus [nt 2265–3094]	2.60×10^−3^ (1.72×10^−3^–3.46×10^−3^)	1989 (1987–1993)	2537 (405–15320)	1.18 (0.64–1.38)	7.05 (6.00–12.96)
Unlinked F locus [nt 2265–2572]	2.69×10^−3^ (1.78×10^−3^–3.54×10^−3^)	1991 (1988–1994)	3176 (651–18028)	1.25 (0.78–1.55)	6.68 (5.37–10.66)
Unlinked B locus [nt 2573–3094]	2.55×10^−3^ (1.91×10^−3^–3.33×10^−3^)	1989 (1986–1992)	2415 (367–13994)	1.13 (0.61–1.26)	7.36 (6.65–13.64)
*gag*					
Linked locus [nt 1087–1560]	1.94×10^−3^ (0.91×10^−3^–3.06×10^−3^)	1988 (1984–1992)	1778 (191–10212)	1.20 (0.59–1.47)	6.93 (5.66–14.10)
Unlinked B locus [nt 1087–1323]	2.02×10^−3^ (0.82×10^−3^–3.24×10^−3^)	1989 (1986–1994)	2092 (322–12273)	1.27 (0.67–1.61)	6.55 (5.17–12.42)
Unlinked F locus [nt 1324–1560]	1.95×10^−3^ (0.85×10^−3^–3.17×10^−3^)	1988 (1983–1991)	1619 (152–9867)	1.15 (0.55–1.42)	7.24 (5.86–15.12)

a Numbers in parentheses are the range of the 95% upper and lower high posterior density. μ, rate of nucleotide substitution; *r*, median rate of exponential growth; λ, epidemic doubling time.

Similarly, we determined the best clock model for estimating the evolution rate of the *gag* gene. The Bayes Factor test of the clock model showed that the lognormal relaxed clock most appropriately described the data, indicating that the substitution rate varies among branches (Table S1). The evolution rate assuming a lognormal relaxed clock was 1.94×10^−3^ substitutions per nucleotide site per year (Table 1). In comparison, previous estimates of the evolution rate of HIV-1 *pol* have ranged from 1.5×10^−3^ to 2.6×10^−3^ substitutions per nucleotide site per year [31], [32], [33], [34]. Our estimate is consistent with the order of magnitude of 10^−3^ expected for the HIV-1 *pol* gene.

To evaluate the possible effect of the recombination breakpoints in the *pol* and *gag* genes on the estimation of nucleotide substitution rate, we applied a multilocus model that assumes different genealogies for each of the unlinked subtype B and subtype F loci [30]. Bayesian MCMC analyses revealed that the estimated substitute rate for the unlinked loci were not significantly different from those of the linked loci. The rates of the unlinked *pol* loci ranged from 2.55×10^−3^ to 2.69×10^−3^ substitutions per nucleotide site per year and those of the unlinked *gag* loci were 1.95×10^−3^ and 2.02×10^−3^ substitutions per nucleotide site per year. Thus the presence of recombination breakpoint in the dataset does not have a significant effect on the evolution rate estimations. Based on the above investigation, the rates of nucleotide substitution for the *pol* (2.60×10^−3^) and *gag* (1.94×10^−3^) genes were used as empirical prior distributions in the subsequent coalescent analyses.

### Demographic history and epidemic parameter of the Brazilian CRF28/29_BF-like HIV-1

**Figure 3:**
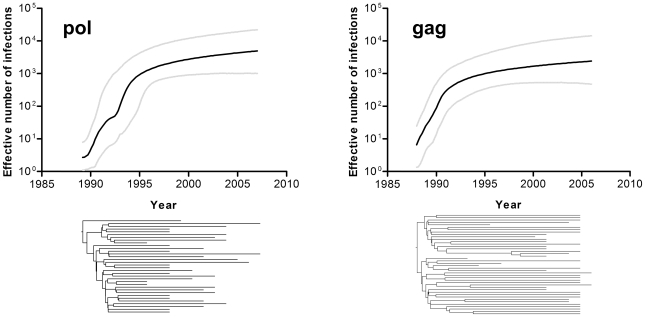
Bayesian skyline plot and phylogenies of the CRF28/29_BF-like HIV-1. Nonparametric reconstruction of the epidemic history with appropriate confidence limits and time-scaled phylogenies of the *pol* and *gag* genes are shown. The demographic history of CRF28/29_BF-like HIV-1 is shown as the median estimate of the effective number of infections through time. The median estimate of the effective number of infections is represented in black, and the 95% confidence limits of the estimate are represented in grey. The trees represent the phylogenetic relationships of sequences belonging to the CRF28/29_BF-like lineage. Both sets of data are shown on the same time scale.

The nonparametric Bayesian skyline method with a lognormal relaxed clock model was used to simultaneously estimate the demographic history and TMRCA of the CRF28/29_BF-like strains. The empirical prior of 2.60×10^−3^ substitutions per nucleotide site per year was used to analyze the *pol* gene. The Bayesian skyline plot of the epidemic history of the CRF28/29_BF-like strains, with appropriate confidence intervals, is shown in Figure 3. The change in the effective number of infections over time appears to follow the model of logistic population growth. A similar observation was inferred from the nonparametric reconstruction of the epidemic history of the CRF28/29_BF-like strains using the *gag* gene and an empirical prior of 1.94×10^−3^ substitutions per nucleotide site per year (Figure 3). We used a Bayes Factor test to determine the likelihoods of the various population growth models. Among the four models tested, the logistic growth model best fit the demographic information contained in both tree topologies thus confirming the epidemic history of the CRF28/29_BF-like strains is best described by the logistic growth model (Table S2).

The evolutionary and demographic parameters associated with the CRF28/29_BF epidemic are shown in Table 1. Our estimates suggest that the CRF28/29_BF lineage originated in the late 1980s. The TMRCA of the CRF28/29_BF-like HIV-1 determined from the *pol* genes was 1989 and that determined from the *gag* genes was 1988. Soon after the emergence of the CRF28/29_BF-like HIV-1, the recombinant virus grew at an exponential rate (Figure 3). The estimated growth rate inferred from the *pol* gene was 1.18 per year, with an epidemic doubling time of seven months. The number of effective infections in 2007 was 2,537. Similar parameters were estimated from the *gag* gene of the CRF28/29_BF-like strains. The inferred growth rate was 1.20 per year, which corresponds to a doubling time of approximately seven months. The number of effective infections in 2006 was 1,778. We then applied the aforementioned nonparametric Bayesian skyline method to the unlinked loci of *pol* and *gag* genes to rule out the possibility that recombination breakpoint may have an effect on estimating the evolutionary and demographic parameters. The resulting estimates for the TMRCA and demographic parameters are listed in Table 1. The TMRCA, growth rate and doubling time estimates are highly consistent among the different linked and unlinked loci of the *pol* or *gag* gene. This confirms previous studies [30], [35] showing that quantitative effect of recombination on evolutionary analyses of HIV-1 may be less severe than initially proposed [28], [29].

## Discussion

In this study, we investigated the evolutionary and demographic histories of the epidemic caused by CRF28_BF and CRF29_BF HIV-1 using a phylogenetic approach and a coalescent framework. Recent reports indicated that BF recombinants have an increased prevalence in the Brazilian population [4], [13], [14], [36], but little is known about the origins of these recombinants. Two of these recombinants, CRF28_BF and CRF29_BF, were detected in 1999 and are prevalent in the southeastern region of Brazil. The southeastern region of the country has been affected by HIV/AIDS very significantly in particular, the state of Sao Paulo, which has the highest number of reported AIDS cases (37% of total cases reported) [37]. Our data showed that the mean TMRCA of CRF28_BF and CRF29_BF was 1988–1989, which was about 10 years after the mean estimated onset date of the subtype F epidemic in Brazil (1976–1981) [10], [18], [38]. In addition, the estimated TMRCA of the CRF28/29_BF-like HIV-1 is consistent with the epidemiological data, which suggested that BF recombinants have been circulating in the Brazilian population since the 1990s. The HIV-1 BF recombinant was initially described in Rio de Janeiro in 1992 [7], [8]. Shortly thereafter, CRF28_BF and CRF29_BF-related recombinants were detected in Rio de Janeiro in 1996 [9]. Multiple cases of viruses sharing the recombination breakpoints of CRF28_BF and CRF29_BF were identified during the period of 1999–2002 [14]. In particular, one of the CRF29_BF samples was isolated in 2002 from a 5-year-old girl who acquired the virus from her mother [4], [14], which suggests that the CRF29_BF was circulating in the population on or before 1997.

Bayesian skyline plots showed that the CRF28/29_BF epidemic follows a model of logistic population growth. Under the logistic model, the effective number of infections grows exponentially from the time of emergence and then decreases in growth toward the present. Soon after the emergence of the CRF28/29_BF-like HIV-1 in 1988–1989, the recombinant virus experienced exponential growth over 5 to 6 years, followed by a decline in its growth rate since 1995. This pattern of population growth is similar to the patterns described for the epidemics of subtype B in the U.S. and the U.K. [31], [39], subtype B in Southeast Asia [40], and subtypes B, C and F in Brazil [11], [18], [41], [42]. In contrast to the aforementioned epidemics, the CRF28/29_BF epidemic has a higher estimated initial growth rate than the initial growth rates of the subtype B epidemic in the U.S. (0.83 year^−1^) and the U.K (0.8 year^−1^), as well as the epidemics of the subtypes B, C and F in Brazil, which ranged from 0.17–0.81 year^−1^. The more rapid initial growth rate of the CRF28/29_BF-like strain indicates that both CRFs harbor a higher transmissibility than their parental subtypes. However, CRF28/29_BF-like strain did not exhibit a strong founder effect and have contributed only to a limited degree to the current epidemic in southeastern Brazil, which is dominated by subtype B. The strong founder effect of CRF07_BC and CRF08_BC in southern China, where both CRFs dominate the epidemics in the region, was probably due to the rapid initial dissemination of HIV-1 in illegal blood donation networks. The absence of such a highly connected transmission network may explain why CRF28_BF and CRF29_BF failed to prevail in the Brazilian AIDS epidemic.

In addition to the BF recombinants, CRF31_BC is circulating in Brazil, mainly in the southern part of the country. Interestingly, several demographic characteristics of CRF28/29_BF and CRF31_BC are remarkably similar. Both epidemics follow a logistic growth model with similar growth rates and doubling times. The growth rates of CRF28_BF and CRF29_BF (1.18–1.20 year^−1^) are very similar to that of CRF31_BC (1.26–1.27 year^−1^), and both have doubling times equivalent to 6–7 months [41]. Both epidemics emerged in the late 1980s, caused an exponentially increasing number of infections during the late 1980s and early 1990s, and resulted in similar numbers of effective infections. The average effective number of infections (i.e., infections that contribute to onward transmission) of CRF28/29_BF is 2,158, which is approximately 6.4% of the infected population. This value is similar to the value of CRF31_BC, where the effective number of infections accounted for about 6.8% of the infected population in Brazil in 2006. Thus, it appears that CRF28/29_BF and CRF31_BC may have similar fitness and transmissibility. Moreover, as with the case of CRF28/29_BF, CRF31_BC also did not dominate the AIDS epidemic in Brazil and has shown a decline in its growth rate in recent years [41].

High-frequency genetic recombination is a hallmark of HIV-1 replication [43], [44], [45], which may lead to a loss of phylogenetic correlation between different loci resulting in overestimations of tree length, evolution rate and TMRCA [28], [29]. Therefore in the current studies, the recombinant locus (CRF28/29_BF-like) and the parental locus (subtype B) were not grouped together in estimating phylodynamic parameters because the two loci have different genealogy and such analysis suffers from the abovementioned biases in estimates on linkage disequilibrium. In contrast, analyzing the phylodynamic relationship of recombinants within the CRF28/29_BF-like clade showed that recombination between the CRFs has no significant effect on the estimated evolution rate, TMRCA and demographic parameters (Table 1). Indeed, previous reports studying the population genetics of CRF07_BC, CRF08_BC and group O HIV-1 have demonstrated similar observations [30], [35]. Moreover, our current investigation together with two recent studies (CRF12_BF in Argentina [34], [46] and CRF38_BF in Uruguay [34]) demonstrated the feasibility of using the recombinant region of CRF genome both for identifying intersubtype recombinant and for phylodynamic analysis of the recombinant. Such framework can simplify and facilitate phylogenetic and population dynamic studies of CRFs.

**Figure 4:**
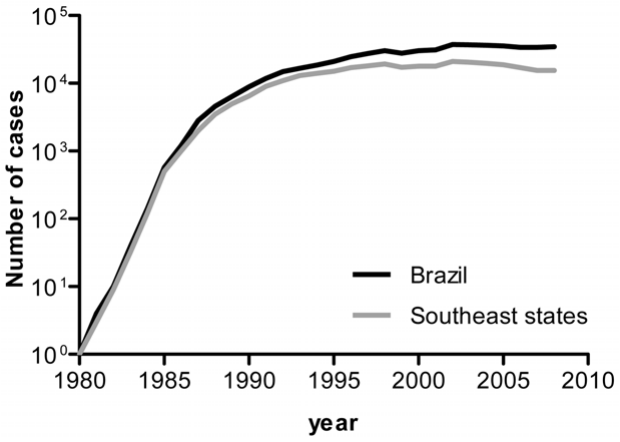
Number of new AIDS cases in Brazil. The number of new AIDS cases reported to the Brazilian Ministry of Health annually during the period 1980–2008. Data were obtained from the Brazilian AIDS cases database (SINAN/SIM/SISCEL). New cases identified in Brazil are represented in black, and those reported in the southeastern states (Espirito Santo, Minas Gerais, Rio de Janeiro and Sao Paulo) are in grey.

Comparing the epidemic history of CRF28/29_BF to the overall epidemiology of AIDS in Brazil, we observed that the exponential growth phase of the BF epidemic coincided with a decline in the reported number of new AIDS cases in Brazil (which reflect epidemics of HIV transmissions occurring several years earlier) (Figure 4). The stabilization of the epidemic in the 1990s may be partly due to the government reform in the mid-1990s, which contributed significantly to the improvement of the healthcare system of the country. This included the availability of HAART in 1996 for people living with HIV and AIDS, which has significantly reduced the number of AIDS cases diagnosed. However, relying on the reported number of new AIDS cases alone and the absence of a comprehensive epidemiological dataset on HIV cases may limit the interpretation of the current investigation and our understanding of the true picture of the HIV/AIDS epidemic in Brazil. This limitation highlights the urgent need for more virological surveillance in the country.

Nonetheless, we observed that the CRF28/29_BF-like strains seemingly did not exert a strong effect on the overall epidemic in Brazil, but appeared to represent a major portion of the BF recombinant population. Of the 67 *pol* and 53 *gag* recombinants of the BF sequences we analyzed, 27 and 35 of them, respectively, were related to CRF28/29_BF. This observation, though, has a caveat: the BF sequences analyzed in this study correspond to data up to 2007, and recent studies have shown that new BF URFs are playing an increasing role in the AIDS epidemic in Brazil [4], [5], [6], [10], [11], [13], [18]. Therefore, with respect to the BF HIV-1 epidemic, although it was largely dominated by the CRF28/29_BF-like strains, it is now likely to be characterized by new CRFs such as CRF39_BF and CRF40_BF, and a variety of recently emerged URFs that do not share a common ancestor [4], [5], [6], [13], [47], [48], [49], [50]. Interestingly, a recent study on the molecular epidemiology of CRF12_BF in Argentina from 1986 to 2008 supports this prediction. The study identified a decreasing prevalence of CRF12_BF and high diversity of BF URFs in Argentina [46]. Therefore, the most recent epidemiological picture of the BF recombinant in Brazil is likely to change and will be revealed when more contemporary BF recombinants are characterized.

In conclusion, we showed that CRF28_BF and CRF29_BF constitute a monophyletic lineage and evolved under similar evolutionary and epidemiological parameters. Both CRFs emerged in the population in the late 1980s, and the rate of spreading of this lineage slowed in the mid-1990s. The diminishing prevalence of CRF28_BF and CRF29_BF combines with high recombination rate between subtype B and subtype F HIV-1 [51]; it is reasonable to assume that new CRFs and URFs will continue to emerge in the Brazilian population. The increasing prevalence of new CRFs and URFs poses a problem in controlling AIDS epidemics given the potential differences among resistance profiles, responses to antiviral interventions and other biological properties of the variants. This highlights the need for functional characterizations of the replication capacity and antiviral susceptibility of HIV-1 recombinants. In addition, more studies detailing the parameters shaping the epidemic and the evolution of HIV-1 recombinants would provide valuable insights into the transmission of HIV-1 in South America and would aid the design of region-specific vaccines.

## Supporting Information

Table S1 **Likelihoods and Bayes Factors of the clock models for the Brazilian CRF28/29_BF-like HIV-1.**
(TIF)

Table S2 **Bayes Factors for testing the best-fit demographic model for the Brazilian CRF28/29_BF-like HIV-1 sequences.**
(TIF)
