# Correction: Analysis of the Origin and Evolutionary History of HIV-1 CRF28_BF and CRF29_BF Reveals a Decreasing Prevalence in the AIDS Epidemic of Brazil

**DOI:** 10.1371/annotation/25854e06-a961-4fec-8664-38667cc278b8

**Published:** 2011-03-17

**Authors:** Natalia Ristic, Jean Zukurov, Wagner Alkmim, Ricardo Sobhie Diaz, Luiz Mario Janini, Mario P. S. Chin

The Supporting Information were incorrectly uploaded as TIFF files. See the correct PDF files here: 

and 
