# Supplementary material for: Analysis of the Origin and Evolutionary History of HIV-1 CRF28_BF and CRF29_BF Reveals a Decreasing Prevalence in the AIDS Epidemic of Brazil
Source: PLoS One. 2011 Mar 1;6(3):e17485. doi: 10.1371/journal.pone.0017485 (PMC3046974; doi:10.1371/journal.pone.0017485)
Supplement: Table S1 — Likelihoods and Bayes Factors of the clock models for the Brazilian CRF28/29_BF-like HIV-1. (TIF) [file pone.0017485.s001.tif]

Table S1. Likelihoods and Bayes Factors of the clock models for the Brazilian CRF28/29_BF-like HIV-1

Clock model	ln P [a]	S.E. +/-	ln (BF) between clock models [b]		
			Strict	Relaxed Exponential	Relaxed Logistic
pol					
Strict	-11908.247	0.275	-	-61.725	-71.351
Relaxed exponential	-11846.522	0.521	61.725	-	-9.626
Relaxed logistic	-11836.896	0.312	71.351	9.626	-
gag					
Strict	-6815.675	0.287	-	-40.153	-42.786
Relaxed exponential	-6775.522	0.256	40.153	-	-2.633
Relaxed logistic	-6772.889	0.267	42.786	2.633	-

[a] Marginal likelihoods estimated according to Suchard et al., 2001, as implemented in the Beast program.

[b] ln (BF): the Bayes Factor is the difference between the marginal likelihoods of the two models under comparison.
