# Supplementary material for: Analysis of the Origin and Evolutionary History of HIV-1 CRF28_BF and CRF29_BF Reveals a Decreasing Prevalence in the AIDS Epidemic of Brazil
Source: PLoS One. 2011 Mar 1;6(3):e17485. doi: 10.1371/journal.pone.0017485 (PMC3046974; doi:10.1371/journal.pone.0017485)
Supplement: Table S2 — Bayes Factors for testing the best-fit demographic model for the Brazilian CRF28/29_BF-like HIV-1 sequences. (TIF) [file pone.0017485.s002.tif]

Table S2. Bayes Factors for testing the best-fit demographic model for the Brazilian CRF28/29_BF-like HIV-1 sequences

Demographic model	ln P [a]	S.E. +/-	ln (BF) between demographic models [b]			
			Constant growth	Exponential growth	Logistic growth	Expansion growth
pol						
Constant growth	-11836.79	0.492	-	17.389	-2.727	9.566
Exponential growth	-11854.18	0.693	-17.389	-	-20.116	-7.823
Logistic growth	-11834.06	0.46	2.727	20.116	-	12.293
Expansion growth	-11846.36	0.58	-9.566	7.823	-12.293	-
gag						
Constant growth	-6774.033	0.552	-	-4.332	-9.694	-1.083
Exponential growth	-6769.701	2.274	4.332	-	-5.362	3.249
Logistic growth	-6764.339	1.397	9.694	5.362	-	8.611
Expansion growth	-6772.95	0.573	1.083	-3.249	-8.611	-

[a] Marginal likelihood estimated according to Suchard et al., 2001, as implemented in the Beast program.

[b] ln (BF): the Bayes Factor is the difference between the marginal likelihoods of two models being compared.
